# Christian Schönbach 1965–2023

**DOI:** 10.1093/bioadv/vbad147

**Published:** 2023-10-20

**Authors:** Anton Kratz, Shoba Ranganathan

**Affiliations:** The Systems Biology Institute, Tokyo 141-0022, Japan; Applied Biosciences, Macquarie University, Sydney, NSW 2109, Australia

**Figure:**
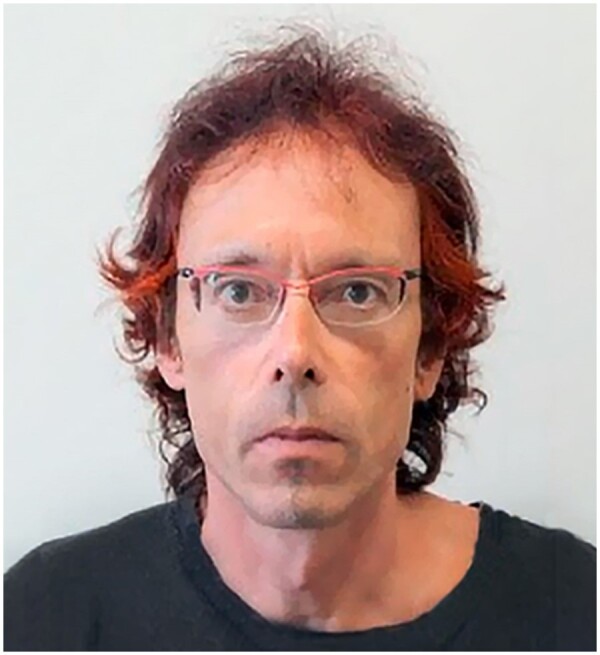
Christian Schönbach, credit: Nazarbayev University, Kazakhstan

‘The comfort of having a friend may be taken away, but not that of having had one’—Seneca

Dr. Christian Schönbach, Chair of the Department of Biology, School of Sciences and Humanities at Nazarbayev University, Kazakhstan, unexpectedly passed away on 15 April 2023. Christian’s contributions to science were immense and span four thematic areas: immunoinformatics, computational and functional genomics, data integration and biological knowledge discovery from databases, and environmental microbiomics. In addition to his research, while serving in leadership roles at the Asia Pacific Bioinformatics Network (APBioNet) and the International Society for Computational Biology (ISCB), he built bridges between diverse scientific communities and enabled the dissemination of scientific knowledge. Christian’s contributions to furthering bioinformatics education and training, in particular in the Asia Pacific region, cannot be overstated. Christian was also a mentor to many students and young researchers.

Christian Schönbach graduated in 1990 from the Julius-Maximilians University Würzburg, Germany, with a master’s degree in biology (Diplom-Biologe) and went on to postgraduate studies in genetics at the Eberhard-Karls University Tübingen, Germany. His contributions to immunology can be traced back to his Ph.D. thesis on the organization of major histocompatibility complex (MHC) class II genes in orangutans (Schönbach 1993). Christian then went to Japan for post-doctoral training in immunology in Prof. Masafumi Takiguchi’s lab at the Department of Tumor Biology, Institute of Biomedical Science, University of Tokyo; embarking on a lifelong journey in East, Southeast, and Central Asia. He continued his immunology research at Chugai Research Institute for Molecular Medicine in Tsukuba, Japan; then moved to Singapore to join the Computational Immunology Group of Kent Ridge Digital Labs (now Institute for Infocomm Research); returned to Japan to join the RIKEN Genomic Sciences Center (GSC) in Yokohama first as a Research Scientist; and then became a Team Leader when starting his own lab in 2002: the Immunoinformatics Research Team at the GSC. During these years, Christian who originally was an experimentalist increasingly utilized computational techniques in his research and pioneered their application to immunology, such as the computational prediction of peptide binding to MHC molecules (Yu *et al.* 2002), applying text mining for vaccine development (Schönbach *et al.* 2004), and integrating immunological data on MHC molecules in the form of a database, FIMM, to aid in vaccine target discovery (Schönbach *et al.* 2005).

**Figure 1.**
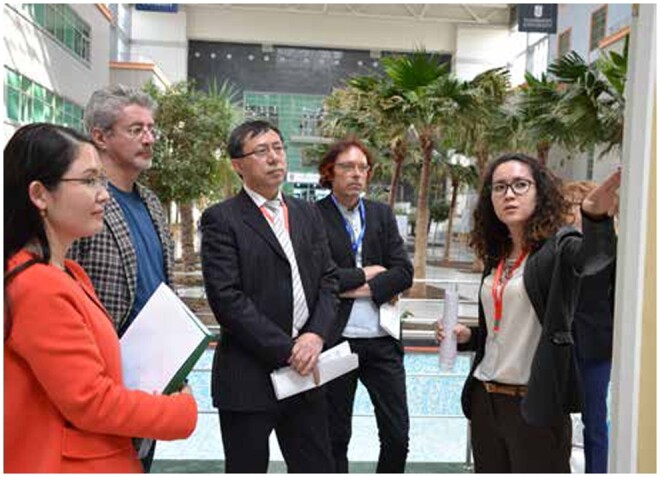
Christian (second from right) at the 2015 ‘Best poster presentation’ for the Young Scientists Award competition in Kazakhstan (credit: https://nla.nu.edu.kz/wp-content/uploads/2016/04/NewsletterVol-5-2015.pdf).

**Figure 2.**
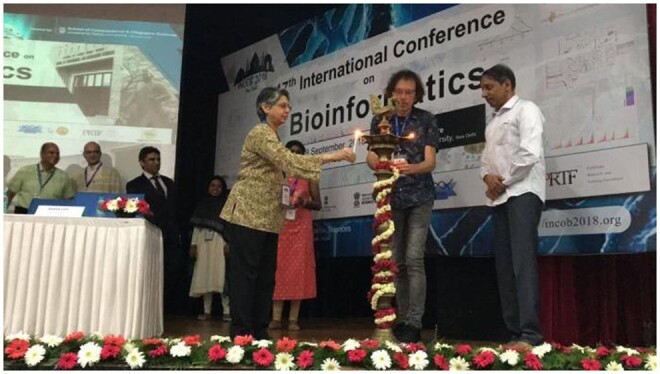
Christian (second from right) inaugurating the 17th International Conference on Bioinformatics, 2018, in New Delhi, India, 2018 as President of Asia Pacific Bioinformatics Network (APBioNet), with Shoba (centre) (credit: https://www.apbionet.org/newsletters/Newsletter_Volume_04_Issue_1_2019.pdf).

In the context of the large, international ‘big data’ research endeavors, Christian’s contributions were critical in the effort to identify all mouse mRNA transcripts (Okazaki *et al.* 2002) and functionally annotate them (Kawai *et al.* 2001) for the Functional Annotation of the Mouse project led by the RIKEN GSC. Christian also pioneered a semi-automated approach to functionally annotate these transcripts (Nagashima *et al.* 2003), continuing his earlier work in immunology, as functional annotation allows the identification of immune-related transcripts, both coding and noncoding (Schönbach 2003) (Figs 1 and 2).

While continuing his work in immunoinformatics and computational and functional genomics, Christian moved into academic roles with an added teaching and mentoring component, first as Associate Professor, Division of Genomics and Genetics, School of Biological Sciences, Nanyang Technological University, Singapore (2006–8), and then as a tenured professor again in Japan at the Department of Bioscience and Bioinformatics, School of Computer Science and Systems Engineering, Kyushu Institute of Technology (2009–13). In 2013, he accepted tenure at the Department of Biology, School of Science and Technology, Nazarbayev University in Kazakhstan. Nazarbayev University had been established only 3 years prior, and Christian was very excited about the rare opportunity to contribute to shaping an entire department and school, and critically influencing the research agenda and curricula, almost from scratch. His research also branched out into a new direction, environmental microbiomics (Meirkhanova *et al.* 2023).

Christian returned to Japan to take up tenure as a full professor at Kumamoto University from 2016 to 2018. In 2018, Christian returned to Kazakhstan, following the demise of his mentor Prof. Akhinori Sarai, this time as the Dean of the Department of Biology in the School of Sciences and Humanities.

Since the early 2010s, Christian made ever-increasing contributions to scientific community service. He served as elected Vice President (2012–6) and then President (2016–20) of APBioNet and as an elected member of the Board of Directors of ISCB since 2018 and tirelessly served as a section editor of *PLoS One* since 2014. A very recent career feature co-authored by Christian describes seven ‘Grand Challenges in bioinformatics education and training’ (Işık *et al.* 2023), which Christian helped identify and make progress toward in his leadership roles in the APBioNet and the ISCB. Christian organized conferences, including APBioNet’s annual meeting, the International Conference on Bioinformatics (InCoB) since 2010, the first GOBLET meeting (Attwood *et al.* 2015), representing APBioNet, and the ICoVax 2012 workshop (He *et al.* 2013), taking a leading role in publishing InCoB proceedings as journal supplements (Schönbach *et al.* 2012, 2014, 2015, 2016), sometimes as joint conferences, such as the 1st ISCB Asia conference in 2011 (Ranganathan *et al.* 2011) and with the Genome Informatics Workshop (Schönbach *et al.* 2015). With these conferences, Christian built bridges not only between sub-disciplines but also between cultures and generations of scientists. One of us (A.K.) remembers how Christian, as the organizer of the InCoB 2010 conference (Ranganathan *et al.* 2010), added a unique touch typical for him by inviting the rock band ‘Negative Selection’ to perform at the conference welcome party and how their music—classic rock—brought the attending scientists together.

Christian’s path took him across East Asia (Japan), Southeast Asia (Singapore), and Central Asia (Kazakhstan), and he moved between these countries multiple times. He took on the difficult tasks of learning Japanese as well as Russian and spearheaded the application of Bioinformatics in the context of the intersection of biology and technology within the Digital Kazakhstan initiative (https://www.youtube.com/watch?v=udedu-So_Hc). Christian thrived conducting his research in these diverse cultural backgrounds, a very rare skill.

In addition to the tremendous contributions to research, teaching, and scientific community service, Christian was also an outstanding mentor. One of us (A.K.) first met Christian in 2005 when doing an internship in his Immunoinformatics Research Team at RIKEN Yokohama. Anton very vividly remembers how Christian clearly identified any issues or roadblocks and immediately set out to find and implement a solution, not only when conducting research but also regarding any organizational issues. Interacting with Christian profoundly influenced his career trajectory. He encouraged Anton to apply for graduate school and set him on the path to become a scientist. After leaving his team, Anton and Christian stayed connected as friends through the years.

Christian’s nurturing of students and young minds is amply evident from the attributes of the 2022 iGEM team from Kazakhstan: ‘We also thank our Department Chair Dr. Christian Schoenbach for ensuring the necessary supervision of the team’s activities, and the safety of our working conditions. Under his guidance, we received generous lab support from our Teaching Assistants Kamilya Sydykova, Nurzhanna Bakuova, and Yerkebulan Yesbolatov who conducted safety sessions with us, prepared all the necessary materials, reagents, and equipment available from the Department, even on weekends and holidays’ (https://2022.igem.wiki/nu-Kazakhstan/attributions).

Christian’s immediate colleagues at Nazarbayev University felt his sudden demise most poignantly and stated ‘His rational and strategic thinking, his attention to detail, and his exceptional organizational skills helped shape today’s Department of Biology and the School. Dr Schoenbach will be sorely missed’ (https://nu.edu.kz/news/in-memory-of-professor-christian-schoenbach).

We know for sure that Christian has advised and mentored many more students and junior scientists throughout his career. Remembering Christian Schönbach, what stands out most in our memories is his contagious energy, his concern for others, and his tireless contributions to science. His passing is an irreparable loss to all of us who knew him.

‘Bandhu sakhe praptasya’—A friend is a treasure acquired.
